# The Influence of Activities and Functional Social Support on Adult Cochlear Implant Outcomes

**DOI:** 10.1097/ONO.0000000000000033

**Published:** 2023-05-12

**Authors:** Julia A. Casazza, Kristen L. Yancey, Jacob B. Hunter

**Affiliations:** 1 Medical School, University of Texas Southwestern Medical Center, Dallas, TX, USA; 2 Department of Otolaryngology-Head and Neck Surgery, University of Texas Southwestern Medical Center, Dallas, TX, USA; 3 Department of Otolaryngology, Thomas Jefferson University, Philadelphia, Pennsylvania

**Keywords:** Aural rehabilitation, Sensorineural hearing loss, Cochlear implantation, Speech perception outcomes, Perceived social support

## Abstract

**Objective:**

The objective of this study is to assess whether patient participation in specific activities and perceived social support correlate with speech perception following cochlear implantation.

**Setting:**

Tertiary referral hospital

**Methods:**

Adult cochlear implantation patients implanted in their poorer hearing ear between January 2019 and December 2020 completed the Functional Social Support Questionnaire (FSSQ) and a modified version of the Victoria Lifestyle Study-Activities Lifestyle Questionnaire (VLS-ALQ). Demographics, FSSQ score, and individual activities were correlated with implanted ear and binaural AzBio scores.

**Results:**

Twenty-three patients completed the survey and had at least 6 months of follow-up with appropriate speech perception testing. The average age at survey completion was 71.7 (SD, 9.1). Average pure-tone average in the contralateral ear was 70.1 (SD: 20) dB. The majority (N = 21, 91.3%) wore a hearing aid in the contralateral ear following cochlear implantation. Mean AzBio_Quiet_ score improvement was 60.6% (range: 20%–99%) in the implanted ear and 42.6% (range: −2% to 67%) binaurally. Work-related social support correlated positively with improvement in the implanted ear (Pearson’s R = 0.473; 95% CI, 0.075-0.741; *P* = 0.023). Improvement in the implanted ear correlated positively with creative writing (R = 0.542; 95% CI, 0.167-0.780; *P* = 0.008), attending films (R = 0.448; 95% CI, 0.044-0.726; *P* = 0.032), going out with friends (R = 0.423; 95% CI, 0.013-0.711; *P* = 0.044) listening to audiobooks (R = 0.433; 95% CI, 0.025-0.717; *P* = 0.039), and public speaking (R = 0.468; 95% CI, 0.069-0.738; *P* = 0.024). Gains in binaural performance correlated positively with watching TV news (R = 0.819; 95% CI, 0.509-0.941; *P* < 0.001) and negatively with eating at restaurants (R = −0.532; 95% CI, −0.829 to −0.002; *P* = 0.05).

**Conclusions:**

Activities that provide intellectual stimulation and engage auditory faculties correlate with greater speech perception testing improvements in adult cochlear implantation patients.

While cochlear implantation is the standard of care for adults with significant sensorineural hearing loss despite appropriately fitted hearing aids, outcomes are highly variable. A multitude of variables have been explored to predict postoperative speech perception, including duration of deafness, age at implantation, electrode array design, cochleostomy type, device use, and insertion depth (1–3). However, little data exist regarding how patient-directed activities may influence outcomes.

There is no standard rehabilitation protocol for adult cochlear implantation recipients, and patients may benefit from specific recommendations to support postimplant performance. Patients sometimes elect formal auditory verbal therapy (AVT), utilize online resources (e.g., angelsound (4)), and/or pursue select activities with their new device (such as streaming audiobooks) (5). Further research into therapies and lifestyle guidance that support adult cochlear implantation rehabilitation remains an unmet need. While several studies have focused on pediatric outcomes, limited data are available examining AVT in adults (6,7). One study found that 10 AVT participants performed at the same level as 7 controls on speech recognition testing 3 and 6 months postimplantation. However, AVT participants partook in only 10 AVT sessions—potentially too few to detect a meaningful difference in outcomes, even with a larger sample size (8). Dornhoffer *et al.* (9) compared passive auditory training (e.g., audiobooks), AVT, and computer-based training in 72 adult cochlear implantation recipients. The use of any rehabilitation technique was associated with increased sentence recognition scores 3 months postimplantation, but the greatest improvements were seen following computer-based training.

While our understanding of the specific activities that assist in cochlear implantation rehabilitation is limited, more engaged patients appear to perform better. In one study of 23 adult cochlear implantation users, the highest scorers on speech perception were patients that intentionally sought out new auditory stimulation and expected that there would be a significant adaptation to their implant (5). Tang *et al*. (10) found relative to patients who lived alone, adult cochlear implantation recipients who lived with another individual scored 20 points higher on AzBio tests. However, the benefits of socialization extend beyond speech perception: among post-lingually deafened cochlear implantation users, 36% of the variance in quality of life (assessed using the International Outcome Inventory—Cochlear Implants) can be explained by patient attitudes and support from others (11). These findings suggest realistic expectations, diverse auditory stimuli, and social interaction are associated with better outcomes.

Motivated to improve patient counseling by highlighting effective habits of successful cochlear implantation users, we sought to understand whether specific lifestyle activities and patients’ self-assessment of their social network correlate with speech perception outcomes. We hypothesized that patients engaged in more lifestyle activities that encourage social interactions would perform better on AzBio sentence testing post-implantation.

## METHODS

The study was approved by the Institutional Review Board (STU 2021-0209). A prospectively maintained cochlear implantation database was used to identify patients implanted between January 1, 2019 and December 31, 2020. Adult cochlear implantation recipients were recruited to complete questionnaires via email with survey links. The estimated time to participate in the study was 20 minutes. All patients were postlingually deafened and were encouraged to wear their implants at all waking hours. Counseling also emphasizes that implant benefits may not be perceived for several months. Patients are also informed of online exercises and offered external AVT resources.

Electronic health records were reviewed to extract demographics, pre- and post-implantation AzBio sentence scores (12), and operative details. Non-English speakers, and patients <18 years were excluded. Study data were collected and managed using REDCap (13,14).

### Patient-Reported Activity Measures

#### Victoria Longitudinal Study-Activities Lifestyle Questionnaire

Victoria Lifestyle Study-Activities Lifestyle Questionnaire (VLS-ALQ) is a 70-item questionnaire that assesses engagement in lifestyle activities in the preceding 2 years (15,16). Originally developed to assess how activity engagement affects cognitive decline, the VLS-ALQ surveys a broad range of social activities but intentionally overrepresents those that require knowledge and intellectual exertion. Each item is graded on a 9-point Likert scale ranging from “never” to “daily,” with higher scores indicating higher activity engagement. We added 11 questions potentially relevant to cochlear implantation rehabilitation to the original VLS-ALQ (Appendix A).

#### Functional Social Support Questionnaire

Functional Social Support Questionnaire (FSSQ) is an 8-item questionnaire that assesses patient perceptions of social support (Appendix B) (17). Respondents indicate their level of satisfaction on a 5-point Likert scale, ranging from “much less than I would like” to “as much as I would like.” Similar to the VLS-ALQ, the FSSQ is scored by summing the responses across all items. Higher scores indicate higher satisfaction.

### Data Analysis

The primary outcome measured was postoperative speech perception improvement with AzBio sentence scores in quiet, obtained at least 6 months following activation. Speech perception was assessed in the implanted ear and binaurally. Improvement was calculated by subtracting the score obtained during cochlear implantation evaluation from the highest postoperative AzBio score in quiet. Pearson’s correlation coefficient (Pearson’s R) was calculated to assess whether demographic variables (age, race, and gender), follow-up, total FSSQ score, and individual items from the VLS-ALQ were associated with speech perception outcomes. *P*-values <0.05 were considered significant. All analyses were performed using R version 4.1.10 (R Foundation for Statistical Computing, Vienna, Austria).

## RESULTS

A total of 154 cochlear implantation patients were emailed survey links, with 53 completed (34% response rate). Patients with less than 6 months of audiometric follow-up, lack of speech perception testing, programming at outside audiology centers, single-sided deafness recipients, and bilateral cochlear implantation were excluded, leaving 23 patients (60.9% male) for analysis. The average age at the time of survey completion was 71.7 ± 9.1 years. The majority (87.0%) of subjects were White. Most (91.3%) patients were implanted with lateral wall electrodes (Table 1). Fifteen (65.2%) met implantation criteria for either ear at evaluation, and 21 (91.3%) wore a hearing aid in the contralateral ear following implantation.

**TABLE 1.** Descriptive statistics and AzBio results for the 23 study participants

Variable (N = 23 unless specified)		
Age, years		71.7 (standard deviation: 9.1)
Gender, n (%)	Male	14 (60.9%)
	Female	9 (39.1%)
Race, n (%)	White	20 (87.0%)
	Other	1 (4.3%)
	Unknown	2 (8.7%)
Ethnicity, n (%)	Hispanic	1 (4.2%)
	Not Hispanic	23 (95.8%)
Electrode type, n (%)	Lateral	21 (91.3%)
	Peri-modiolar	2 (8.7%)
Mean (SD) pure tone average, contralateral ear, decibels		70.1 (20.0)
Mean (SD) preimplantation Azbio_Quiet_		
Binaural (N = 14)		46.3 (20.7%)
Ear to be implanted		23.1% (24.1%)
Contralateral		50.4% (28.2%)
Mean (SD) postimplantation improvement in AzBio_Quiet_		
Mean (binaural) (N = 14)		42.6% (18.6%)
Mean implanted ear		60.6% (24.1%)
Mean (SD) duration of audiometric follow-up, years		
Implanted ear		1.154 (0.510)
Binaural (N = 14)		1.108 (0.461)

### Patient-reported Activities and Speech Perception Outcomes in the Implanted Ear

Neither age at initial evaluation (R = –0.200; 95% CI, –0.566 to 0.230; *P* = 0.359) nor the length of follow-up (R = 0.260; 95% CI, –0.170 to 0.670; *P* = 0.231) correlated with improvement in AzBio scores. While the total FSSQ score was not correlated (R = 0.376; 95% CI, –0.043 to 0.682; *P* = 0.077), higher responses to 1 question, “I get a chance to talk to others about problems at work or with my housework,” correlated with improvement in ipsilateral AzBio scores (R = 0.472; 95% CI, 0.075-0.741; *P* = 0.022). Thus, patients who feel more supported at work performed better.

Multiple items on the VLS-ALQ were correlated with gains in speech perception in the implanted ear (Table 2). These activities included engagement in creative writing (R = 0.542; 95% CI, 0.167-0.780; *P* = 0.008), attending films (R = 0.448; 95% CI, 0.044-0.726; *P* = 0.032), going out with friends (R = 0.423; 95% CI, 0.013-0.711; *P* = 0.044), public speaking (R = 0.468; 95% CI, 0.069-0.738; *P* = 0.024), and listening to audiobooks (R = 0.433; 95% CI, 0.025-0.717; *P* = 0.039).

**TABLE 2.** Correlation between patient-reported lifestyle activities and improvement on AzBio in quiet in the implanted ear (n = 23)

	Pearson’s R (95% CI)	*P*-value
Household repairs (i.e. painting)	−0.247 (−0.598 to 0.184)	0.255
Mechanical repairs (i.e. car, lawn mower)	−0.132 (−0.516 to 0.296)	0.548
Purchase item requiring assembly	0.006 (−0.407 to 0.417)	0.978
Woodworking, carpentry, furniture refurnishing	0.191 (−0.251 to 0.567)	0.395
Playing a musical instrument	−0.237 (−0.599 to 0.204)	0.287
**Creative writing**	**0.542 (0.167-0.780**)	**0.008**
Photography	0.279 (−0.150 to 0.620)	0.197
Collecting items	−0.180 (−0.551 to 0.251)	0.411
Sewing	0.285 (−0.144 to 0.624)	0.187
Gardening	0.267 (−0.163 to 0.617)	0.219
Exercising	0.095 (−0.330 to 0.488)	0.666
Recreational sports (i.e. tennis, bowling, and golf)	−0.022 (−0.431 to 0.393)	0.919
Aerobic exercise	0.082 (−0.341 to 0.478)	0.709
Flexibility training	0.412 (0-7.047)	0.051
Strength training	0.168 (−0.262 to 0.543)	0.444
Solving word puzzles	−0.080 (−0.486 to 0.353)	0.722
Playing card games	0.059 (−0.361 to 0.461)	0.786
Solving jigsaw puzzles	−0.137 (−0.520 to 0.292)	0.534
Playing board games	0.218 (−0.213 to 0.578)	0.320
Playing knowledge games	−0.032 (−0.0438 to 0.386)	0.886
Playing word games	0.188 (−0.243 to 0.557)	0.391
Reading newspapers	−0.011 (−0.421 to 0.403)	0.960
Reading books/magazines for leisure	0.352 (−0.070 to 0.667)	0.099
Reading for work	0.166 (−0.264 to 0.541)	0.448
Going to the library	−0.111 (−0.500 to 0.316)	0.614
Watching TV news	0.063 (−0.359 to 0.463)	0.775
Watching documentaries	0.287 (−0.142 to 0.625)	0.185
Watching game shows	0.073 (−0.350 to 0.471)	0.741
Watching comedy	0 (−0.412 to 0.414)	0.999
Writing a letter	0.285 (−0.144 to 0.624)	0.188
Using software on a computer	0.257 (−0.174 to 0.605)	0.237
Using a calculator	0.261 (−0.169 to 0.608)	0.229
Preparing income taxes	0.056 (−0.365 to 0.458)	0.799
Doing math	0.169 (−0.262 to 0.543)	0.441
**Attending films**	**0.448 (0.044 to 0.726**)	**0.032**
Attending a public lecture	0.096 (−0.330 to 0.487)	0.664
Eating at a restaurant	−0.004 (−0.415 to 0.409)	0.984
Talking on the phone	0.178 (−0.253 to 0.550)	0.417
Visiting friends	0.322 (−0.104 to 0.648)	0.134
**Going out with friends**	**0.423 (0.013 to 0.711**)	**0.044**
Attending parties	0.152 (−0.278 to 0.531)	0.489
Hosting dinners or parties	0.357 (−0.065 to 0.670)	0.095
Attending church	0.162 (−0.268 to 0.538)	0.460
Praying	0.135 (−0.294 to 0.518)	0.540
Attending club meetings	0.369 (−0.051 to 0.678)	0.083
Attending organized social events	0.209 (−0.222 to 0.572)	0.339
Participating in political activities	0.077 (−0.346 to 0.474)	0.727
**Giving a public talk/lecture**	**0.468 (0.069 to 0.738**)	**0.024**
Volunteering	0.318 (−0.109 to 0.645)	0.140
Engaging in business activities	0.085 (−0.339 to 0.480)	0.701
On-the-job training	0.350 (−0.03 to 0.666)	0.102
Taking college classes	0.359 (−0.063 to 0.672)	0.092
Learning/practicing a foreign language	0.080 (−0.343 to 0.477)	0.717
Domestic travel	0.370 (−0.050 to 0.679)	0.082
Travel within Texas	0.047 (−0.372 to 0.451)	0.832
Travel abroad	−0.128 (−0.513 to 0.300)	0.561
Walking	−0.238 (−0.592 to 0.193)	0.274
Swimming	0.016 (−0.399 to 0.425)	0.942
Bicycling	0.240 (−0.191 to 0.594)	0.270
Dancing	0.042 (−0.377 to 0.445)	0.848
Listening to podcasts	0.378 (−0.040 to 0.684)	0.075
**Listening to audiobooks**	**0.433 (0.025 to 0.717**)	**0.039**
Listening to classical music	0.265 (−0.165 to 0.610)	0.222
Listening to favorite genre of music	0.317 (−0.109 to 0.645)	0.140
Listening to talk radio	0.179 (−0.252 to 0.551)	0.414
Spending time with children	−0.024 (−0.432 to 0.392)	0.912
Spending time with grandchildren	0.201 (−0.278 to 0.600)	0.408
Spending time with great-grandchildren	−0.189 (−0.731 to 0.500)	0.601

Significant correlations are bolded.

### Patient-reported Activities and Binaural Speech Perception Outcomes

Of the 14 patients for whom binaural results were available, 12 (85.7%) met the criteria for implantation in the other ear at initial evaluation, and 13 (92.9%) wore a hearing aid in the nonimplanted ear after cochlear implantation. Neither age at initial evaluation (R = 0.203; 95% CI, –0.367 to 0.662; *P* = 0.486) nor duration of follow-up (R = –0.357; 95% CI, –0.746 to 0.214; *P* = 0.210) correlated with improvement in binaural AzBio scores. Total FSSQ score did not correlate with improvement in AzBio scores (R = 0.181; 95% CI, –0.387 to 0.649; *P* = 0.536).

Pearson’s correlation between VLS-ALQ responses and the postimplantation improvement in binaural AzBio_Quiet_ are shown in Table 3. There was a strong positive correlation between watching TV news (R = 0.819; 95% CI, 0.509-0.941; *P* < 0.001) and improvement in AzBio scores. There was a strong negative correlation between eating at restaurants (R = –0.532; 95% CI, –0.829 to –0.002; *P* = 0.050) and improvement in binaural AzBio scores.

**TABLE 3.** Correlation between patient-reported lifestyle activities and binaural AzBio_Quiet_ improvement following cochlear implantation (n = 14)

	Pearson’s R (95% CI)	*P*-value
Household repairs (i.e. painting)	−0.251 (−0.690 to 0.322)	0.387
Mechanical repairs (i.e. car, lawn mower)	−0.266 (−0.698 to 0.308)	0.358
Purchase item requiring assembly	−0.163 (−0.638 to 0.403)	0.588
Woodworking, carpentry, furniture refurnishing	0.115 (−0.443 to 0.608)	0.696
Playing a musical instrument	−0.049 (−0.564 to 0.495)	0.870
Creative writing	−0.132 (−0.619 to 0.429)	0.654
Photography	0.007 (−0.526 to 0.536)	0.981
**Collecting items**	**−0.693** (−**0.0895** to −**0.257**)	**0.006**
Sewing	0.336 (−0.237 to 0.735)	0.241
Gardening	0.088 (−0.464 to 0.591)	0.765
Exercising	0.086 (−0.466 to 0.590)	0.770
Recreational sports (i.e. tennis, bowling, and golf)	0.186 (−0.382 to 0.652)	0.524
Aerobic exercise	−0.203 (−0.663 to 0.367)	0.486
Flexibility training	−0.058 (−0.571 to 0.488)	0.845
Strength training	−0.106 (−0.603 to 0.450)	0.718
Solving word puzzles	0.150 (−0.414 to 0.631)	0.610
Playing card games	0.330 (−0.244 to 0.732)	0.250
Solving jigsaw puzzles	−0.097 (−0.596 to 0.457)	0.742
Playing board games	0.217 (−0.355 to 0.670)	0.457
Playing knowledge games	0.224 (−0.348 to 0.674)	0.442
Playing word games	0.038 (−0.503 to 0.557)	0.898
Reading newspapers	0.111 (−0.446 to 0.606)	0.706
Reading books/magazines for leisure	0.348 (−0.224 to 0.742)	0.222
Reading for work	−0.419 (−0.777 to 0.144)	0.136
Going to the library	0.348 (−0.223 to 0.742)	0.222
**Watching TV news**	**0.819 (0.509 to 0.941**)	**<0.001**
Watching documentaries	0.385 (−0.183 to 0.760)	0.174
Watching game shows	−0.089 (−0.591 to 0.464)	0.763
Watching comedy	−0.353 (−0.744 to 0.218)	0.215
Writing a letter	0.211 (−0.360 to 0.667)	0.468
Using software on a computer	−0.045 (−0.562 to 0.498)	0.880
Using a calculator	−0.042 (−0.560 to 0.500)	0.887
Preparing income taxes	−0.407 (−0.771 to 0.158)	0.149
Doing math	0.055 (−0.489 to 0.569)	0.851
Attending films	0.098 (−0.457 to 0.597)	0.740
Attending a public lecture	0.131 (−0.430 to 0.618)	0.656
**Eating at a restaurant**	**−0.532** (**−0.829** to **−0.002**)	**0.050**
Talking on the phone	−0.314 (−0.724 to 0.259)	0.274
Visiting friends	0.200 (−0.370 to 0.660)	0.494
Going out with friends	0.287 (−0.288 to 0.709)	0.320
Attending parties	−0.084 (−0.589 to 0.467)	0.773
Hosting dinner or parties	−0.029 (−0.551 to 0.509)	0.921
Attending church	0.001 (−0.530 to 0.532)	0.996
Praying	−0.02 (−0.551 to 0.510)	0.923
Attending club meetings	0.026 (−0.511 to 0.550)	0.929
Attending organized social events	0.153 (−0.411 to 0.632)	0.601
Participating in political activities	0.199 (−0.371 to 0.660)	0.496
Giving a public talk/lecture	0.197 (−0.372 to 0.659)	0.499
Volunteering	0.264 (−0.310 to 0.697)	0.362
Engaging in business activities	−0.356 (−0.746 to 0.215)	0.212
On-the-job training	−0.147 (−0.628 to 0.416)	0.617
Taking college classes	−0.040 (−0.559 to 0.501)	0.892
Learning/practicing a foreign language	0.038 (−0.502 to 0.557)	0.898
Domestic travel	0.117 (−0.441 to 0.610)	0.691
Travel within Texas	−0.036 (−0.556 to 0.504)	0.904
Travel abroad	0.201 (−0.369 to 0.661)	0.491
Walking	−0.448 (−0.791 to 0.108)	0.108
Swimming	−0.345 (−0.739 to 0.229)	0.229
Bicycling	0.072 (−0.476 to 0.581)	0.805
Dancing	0.025 (−0.512 to 0.548)	0.932
Listening to podcasts	−0.148 (−0.630 to 0.415)	0.612
Listening to audiobooks	−0.046 (−0.563 to 0.497)	0.876
Listening to classical music	0.421 (−0.141 to 0.778)	0.134
Listening to favorite genre of music	0.502 (−0.39 to 0.815)	0.0675
Listening to talk radio	0.297 (−0.277 to 0.715)	0.302
Spending time with children	0.183 (−0.385 to 0.650)	0.532
Spending time with grandchildren	−0.231 (−0.711 to 0.400)	0.471
Spending time with great-grandchildren	0.023 (−0.743 to 0.763)	0.961

Significant correlations are bolded.

## DISCUSSION

To our knowledge, this is the first study to assess how patient-reported engagement in lifestyle activities correlates to cochlear implantation outcomes. Creative writing, attending films, going out with friends, public speaking, and listening to audiobooks correlated positively with improvement in the implanted ear, and watching TV news correlated with improvement in the binaural condition. While social engagements with friends influenced outcomes, functional social support (as assessed by the FSSQ; Appendix B) did not. This result suggests that actual participation in social activities—particularly those that engage auditory processing skills—rather than subjective feelings of social connection, are more important for improved speech perception outcomes following cochlear implantation.

Multiple explanations may account for the link between social engagement and improved cochlear implantation outcomes. It is possible that patients who are more active socially are healthier overall, allowing the time and energy needed for successful auditory rehabilitation. Additionally, if the relationship between social engagement and better speech perception outcomes is causative, perhaps social interactions employ cognitive resources in a manner that facilitates adaptation to hearing with cochlear implantation or engages resources that leads to improved outcomes. Social engagement might preserve gray matter volume in areas associated with sensory processing, language, and memory, which are shown to be associated with improved outcomes (18). Patients with more active social lives may also have greater cause to use their cochlear implantation and thus invest more heavily in adapting to them. Further study is needed to untangle the connection between socialization, cognitive aging, and adaptation to cochlear implantation.

Consistent with prior reports, participation in intellectually stimulating activities correlates with greater gains following cochlear implantation. Dornhoffer *et al.* (19) found the use of audiobooks is associated with increased improvement in speech perception and quality of life in adult cochlear implantation patients. Notably, verbal working memory, but not processing speed or intellectual capacity, predicts 6-month speech recognition AzBio scores, in adult cochlear implantation patients (20). Perhaps verbally/linguistically-intensive activities such as creative writing, public speaking, and listening to audiobooks support speech perception by strengthening verbal working memory.

Surprisingly, the variables correlating with greater improvement in postimplantation binaural speech perception differed from the implanted ear. For example, watching TV news was positively correlated, and eating at restaurants correlated negatively with greater improvement in binaural speech perception. This discrepancy between the ipsilateral and binaural analyses could result from fewer available data for the binaural condition (binaural AzBio scores were available for 14 patients versus 23 for the implanted ear), which could skew results.

There are several limitations to our study. The retrospective nature of our study means it is subject to recall bias. Controlled trials are also needed to determine whether the relationships between activities and postimplantation speech perception scores are causative. While the VLS-ALQ inventories a range of activities, it is possible there are activities patients engage in to help them adapt to cochlear implantation that were not queried. We elected to not apply commonly used statistical approaches (e.g., Bonferroni or Benjamini-Hochberg Correction) to control for multiple comparisons, as these assume the individual items tested are independent of each other. We anticipated that patient involvement in certain types of similar activities would cluster together. Our sample size was also relatively small and lacked diversity, limiting statistical power and generalizability. Device use was also not available for most study participants and was not included in the analysis. Additionally, as the period over which patients were queried overlapped with the Coronavirus Disease 19 (COVID-19) pandemic, it is also likely that participation in work and leisure activities was curtailed in ways we cannot fully adjust for. Indeed, Knickerbocker *et al.* (21) found that adults implanted during the COVID-19 pandemic improved less between pre-operative and 6-month AzBio scores than those implanted just prior, likely due to reduced daily use and fewer social interactions.

## CONCLUSION

Programs to support newly implanted cochlear implantation patients in reaching their aural rehabilitation potential are needed. Our results suggest that a deeper investigation into the kinds of social interactions patients have and an assessment of how they communicate during these interactions are worth pursuing. We found that creative writing, attending films, public speaking, and going out with friends positively correlated with improvements in speech perception in the implanted ear. Watching TV news positively correlated with, and eating out at restaurants negatively correlated with improvements in binaural speech perception. These findings highlight social and intellectual engagement as potential targets for cochlear implantation rehabilitation.

## FUNDING SOURCES

Internal departmental funding was utilized without commercial sponsorship or support.

## CONFLICT OF INTEREST

None declared.

## DATA AVAILABILITY

The datasets generated during and/or analyzed during the current study are not publicly available but are available from the corresponding author on reasonable request.

## References

[1] Naples JG Ruckenstein MJ. Cochlear implant. Otolaryngol Clin North Am. 2020;53:87–102. 31677740 10.1016/j.otc.2019.09.004

[2] Snels C Inthout J Mylanus E Huinck W Dhooge I. Hearing preservation in cochlear implant surgery: a meta-analysis. Otol Neurotol. 2019;40:145–153. 30624395 10.1097/MAO.0000000000002083

[3] O’Connell BP Hunter JB Haynes DS. Insertion depth impacts speech perception and hearing preservation for lateral wall electrodes. Laryngoscope. 2017;127:2352–2357. 28304096 10.1002/lary.26467 PMC5825186

[4] Angel Sound - Interactive Listening Rehabilitation and Functional Hearing Test Program. Emily Fu Foundation. Available at: http://angelsound.tigerspeech.com/. Accessed January 7, 2023.

[5] Harris MS Capretta NR Henning SC Feeney L Pitt MA Moberly AC. Postoperative rehabilitation strategies used by adults with cochlear implants: a pilot study. Laryngoscope Investig Otolaryngol. 2016;1:42–48. 10.1002/lio2.20 PMC5510267 28894803

[6] Percy-Smith L Tønning TL Josvassen JL. Auditory verbal habilitation is associated with improved outcome for children with cochlear implant. Cochlear Implants Int. 2017;19:38–45. 29058555 10.1080/14670100.2017.1389020

[7] Thomas ES Zwolan TA. Communication mode and speech and language outcomes of young cochlear implant recipients: a comparison of auditory-verbal, oral communication, and total communication. Otol Neurotol. 2019;40:e975E975–e975e983. 31663992 10.1097/MAO.0000000000002405

[8] Baungaard LH Sandvej MG Krøijer JS. Auditory verbal skills training is a new approach in adult cochlear implant rehabilitation. Dan Med J. 2019;66:A5535. Available at: https://pubmed.ncbi.nlm.nih.gov/30864546/. Accessed August 26, 2021. 30864546

[9] Dornhoffer JR Reddy P Ma C Schvartz-Leyzac KC Dubno JR McRackan TR. Use of Auditory Training and Its Influence on Early Cochlear Implant Outcomes in Adults. Otol Neurotol. 2022;43:e165–e173. 10.1097/MAO.0000000000003417 PMC8752503 34772887

[10] Tang L Thompson CB Clark JH Ceh KM Yeagle JD Francis HW. Rehabilitation and psychosocial determinants of cochlear implant outcomes in older adults. Ear Hear. 2017;38:663–671. 28542018 10.1097/AUD.0000000000000445

[11] Hallberg LR Ringdahl A Holmes A Carver C. Psychological general well-being (quality of life) in patients with cochlear implants: importance of social environment and age. Int J Audiol. 2005;44:706–711. 16450922 10.1080/14992020500266852

[12] Spahr AJ Dorman MF. Performance of subjects fit with the advanced bionics CII and nucleus 3G cochlear implant devices. Arch Otolaryngol Head Neck Surg. 2004;130:624–628. 15148187 10.1001/archotol.130.5.624

[13] Harris PA Taylor R Thielke R Payne J Gonzalez N Conde JG. Research electronic data capture (REDCap)—a metadata-driven methodology and workflow process for providing translational research informatics support. J Biomed Inform. 2009;42:377–381. 18929686 10.1016/j.jbi.2008.08.010 PMC2700030

[14] Harris PA Taylor R Minor BL; REDCap Consortium. The REDCap consortium: building an international community of software platform partners. J Biomed Inform. 2019;95:103208. 31078660 10.1016/j.jbi.2019.103208 PMC7254481

[15] Hultsch DF Hammer M Small BJ. Age differences in cognitive performance in later life: relationships to self-reported health and activity life style. Journals Gerontol. 1993;48:P1–P11. 10.1093/geronj/48.1.p1 8418144

[16] Shaikh K. Development and validation of the memory impact questionnaire. 2016.

[17] Broadhead WE Gehlbach SH de Gruy FV Kaplan BH. The Duke-UNC functional social support questionnaire: measurement of social support in family medicine patients. Med Care. 1988;26:709707–709723. 10.1097/00005650-198807000-00006 3393031

[18] Sun Z Won J Hong S. Cortical reorganization following auditory deprivation predicts cochlear implant performance in postlingually deaf adults. Hum Brain Mapp. 2021;42:233–244. 33022826 10.1002/hbm.25219 PMC7721232

[19] Dornhoffer JR Reddy P Ma C Schvartz-Leyzac KC Dubno JR McRackan TR. Use of auditory training and its influence on early cochlear implant outcomes in adults. Otol Neurotol. 2022;43:E165–E173. 34772887 10.1097/MAO.0000000000003417 PMC8752503

[20] Heydebrand G Hale S Potts L Gotter B Skinner M. Fax +41 61 306 12 34 E-Mail karger@karger.ch cognitive predictors of improvements in adults’ spoken word recognition six months after cochlear implant activation. Audiol Neurotol. 2007;12:254–264. 10.1159/000101473 17406104

[21] Knickerbocker A Bourn S Goldstein MR Jacob A. Cochlear implant outcomes in elderly recipients during the COVID-19 pandemic. Otol Neurotol. 2021;42:e1256–e1262. 34267095 10.1097/MAO.0000000000003291 PMC8443422

